# A Case of Mammary-Type Myofibroblastoma Occurring in the Inguinal Region

**DOI:** 10.7759/cureus.62679

**Published:** 2024-06-19

**Authors:** Koki Kawakami, Yoshitoshi Sato, Shinichi Sugimoto

**Affiliations:** 1 Department of Surgery, Unnan City Hospital, Shimane, JPN; 2 Department of Surgery, Matsue Red Cross Hospital, Shimane, JPN

**Keywords:** case report, benign tumor, inguinal hernia, inguinal tumor, mammary-type myofibroblastoma

## Abstract

Mammary-type myofibroblastoma (MTMF) is a rare benign tumor of mesenchymal cells that occurs in the inguinal region, abdominal wall, buttocks, back, and vaginal wall. When a soft tissue mass develops in the inguinal region, there may be a risk of misdiagnosing it as an inguinal hernia, especially if it is asymptomatic. We report a rare case of mammary-type myofibroblastoma occurring in the inguinal region. The patient was a 40-year-old male who noticed swelling in the right inguinal region two years prior and consulted a nearby medical clinic. He was diagnosed with a right inguinal hernia and referred to our hospital. On physical examination, a protrusion was observed in the right inguinal region, and due to difficulty in reduction, emergency surgery was performed, suspecting intestinal incarceration. Intraoperatively, no inguinal hernia was found, but a mobile yellowish mass was identified. There were no malignant features, and the mass was excised. The pathological examination revealed mammary-type myofibroblastoma. When we examine a patient with a complaint of inguinal swelling, it is important to consider not only inguinal hernia but also other conditions such as soft tissue tumors.

## Introduction

Mammary-type myofibroblastoma (MTMF) is a rare benign tumor of mesenchymal cells that occurs in the inguinal region, abdominal wall, buttocks, back, and vaginal wall [1-3]. As for the etiology, some hormonal abnormalities have been implicated. When a soft tissue tumor develops in the inguinal region, there may be cases where it is mistakenly diagnosed as an inguinal hernia, especially when asymptomatic [4]. Additionally, other diseases, such as aneurysms, varicose veins, endometriosis, and hydrocele of the canal of the nuck, require a differential diagnosis from an inguinal hernia. In this paper, we report a case where the preoperative diagnosis was inguinal hernia, but postoperatively, it was found to be MTMF.

## Case presentation

**Figure 1:**
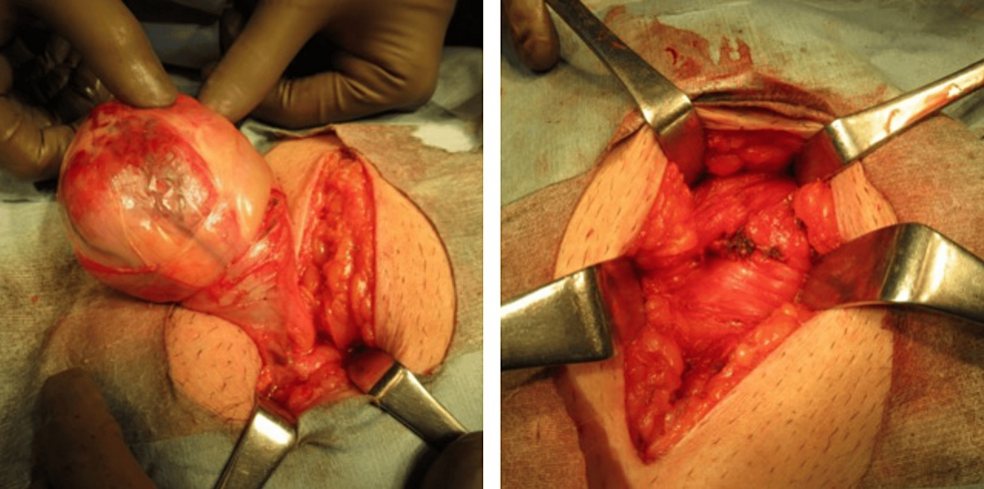
Intraoperative findings Rubbery to firm mass measuring about 8 cm was observed. It was dissected from the surrounding tissues, and nutrient vessels branching from the inside were ligated before excising the mass.

**Figure 2:**
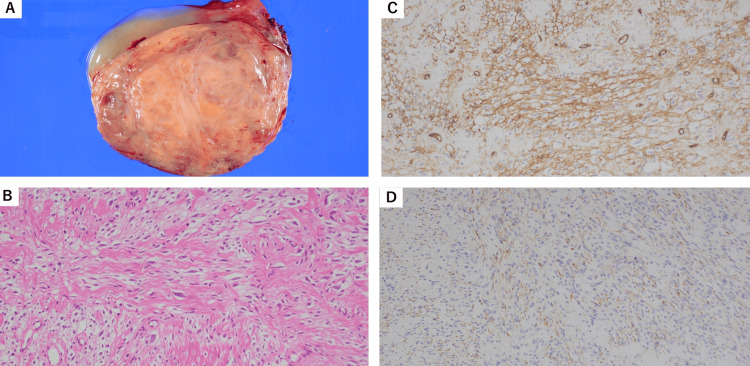
Pathological findings (A) The cut surface of the specimen exhibited a yellowish hue with fibrous septa structures accompanied by mucoid changes. (B) Spindle-shaped cells with mild nuclear enlargement proliferated in fascicular and storiform patterns (×400). (C,D) Immunohistochemically, spindle-shaped cells were positive for CD34, Desmin, and ER (×400).

A 40-year-old male presented with noticed progressively enlarging swelling in the right inguinal region for two years. He was diagnosed with a right inguinal hernia and referred to our hospital. On the physical examination, a protrusion was observed in the right inguinal region. Due to difficulty in reduction, emergency surgery was performed for suspicious intestinal incarceration without imaging. We made an 8-cm incision in the right inguinal region. Initially, it was thought that the small intestine was incarcerated in the right inguinal hernia. However, there was no inguinal hernia, and a well-defined, rubbery-to-firm mass measuring about 8 cm was observed in the subcutaneous tissue. It was dissected from the surrounding tissues, and vessels branching from the fatty tissue were ligated before excising the mass. The tumor was believed to originate from the surrounding fatty tissue rather than the spermatic cord. The cut surface of the mass is yellowish with fibrous septa and focal mucoid changes (Figure 1). Histologically, this is a proliferation of spindle cells with mild nuclear enlargement in fascicular and storiform patterns. Interspersed fat cells, areas with mucoid and edematous changes, and collagen fibers were observed. Immunohistochemically, these spindle cells were positive for CD34, Desmin, and the estrogen receptor (Figure 2). The final diagnosis was confirmed to be MTMF. The surgical margins were not involved. The patient received no further treatment and is alive with no evidence of recurrence or metastasis 24 months after surgery.

## Discussion

Wargotz et al. first reported MTMF in 1987 as a benign tumor of mesenchymal cell origin occurring within the mammary gland [5]. In 2001, McMenamin and Fletcher reported a histologically identical tumor occurring outside the mammary gland as MTMF, leading to the expansion of the concept of this disease [1]. MTMF predominantly affects middle-aged to older men and occurs in the inguinal region, abdominal wall, buttocks, back, and vaginal wall [1-3]. It is painless and tends to grow slowly. Reports commonly come from breast surgery and urology departments, making it a less familiar condition for gastrointestinal surgeons.

On gross findings, it is a well-circumscribed, not encapsulated, solid lump with a firm, whitish-gray nodular or whorled-cut surface. Sometimes, it may be multilobulated [3].

Histopathologically, a well-defined lesion without capsules is observed in the subcutaneous tissue. Thin fibroblasts are present, surrounded by thin collagen fibers and scattered fat cells. Immunohistochemical staining shows positivity for vimentin, desmin, ER, PR, AR, BCL2, and CD10. Cytokeratins S100, p63, and Rb lack immunohistochemical staining [6,7].

When a soft tissue tumor develops in the inguinal region, it may be mistakenly diagnosed as an inguinal hernia, especially when asymptomatic. Particularly, characteristic imaging findings of MTMF have not been reported, making preoperative diagnosis difficult [4,8]. This is thought to be due to variations in the amount of intervening adipose tissue in this condition, leading to differences in MRI findings among cases. While needle biopsy and intraoperative frozen section examination may aid in diagnosis, surgical excision is usually necessary for a definitive diagnosis in most cases [9].

There is debate about whether to routinely perform imaging studies such as abdominal CT scans before surgery for clinically suspecting inguinal hernia. The specificity of diagnosis based solely on physical examination findings for inguinal hernia is 70-90% [10]. In cases where typical symptoms (inguinal swelling upon standing or increased intra-abdominal pressure) are present and manual reduction is easily achieved, surgery for inguinal hernia may be considered without imaging studies. Ultrasonography shows a uniform hypoechoic area in the case of hydrocele testis but a heterogeneous hyperechoic area in the case of liposarcoma. In addition, MRI shows high signal intensity on T1-weighted images in the case of a fat-fitting inguinal hernia and lipoma and a low signal intensity in the case of liposarcoma [11]. When there are no typical symptoms, such as in this case, imaging studies should be performed to guide treatment decisions. Differential diagnosis of inguinal swelling includes a wide range of conditions such as aneurysms, varicose veins, soft tissue tumors, and Nuck duct cysts, each requiring different treatment approaches. Therefore, careful judgment is necessary.

## Conclusions

In summary, we presented a case of MTMF in a 40-year-old male, and we reviewed the English and Japanese literature. Rare tumors, such as MTMF, may be present in inguinal swellings that differ from the typical symptoms of inguinal hernia. Preoperative investigations, such as imaging studies, were considered necessary in the case of atypical inguinal swellings.
